# Nucleotide Sequencing and SNP Detection of Toll-Like Receptor-4 Gene in Murrah Buffalo (*Bubalus bubalis*)

**DOI:** 10.5402/2012/659513

**Published:** 2012-03-29

**Authors:** M. Mitra, S. Taraphder, G. S. Sonawane, A. Verma

**Affiliations:** ^1^Department of Animal Genetics and Breeding, Faculty of Veterinary and Animal Sciences, West Bengal University of Animal and Fishery Sciences, 37768 Kshudiram Bose Sarani, West Bengal, Kolkata 700037, India; ^2^Dairy Cattle Breeding Division, NDRI, Karnal-132001, Haryana, India

## Abstract

Toll-like receptor-4 (TLR-4) has an important pattern recognition receptor that recognizes endotoxins associated with gram negative bacterial infections. The present investigation was carried out to study nucleotide sequencing and SNP detection by PCR-RFLP analysis of the TLR-4 gene in Murrah buffalo. Genomic DNA was isolated from 102 lactating Murrah buffalo from NDRI herd. The amplified PCR fragments of TLR-4 comprised of exon 1, exon 2, exon 3.1, and exon 3.2 were examined to RFLP. PCR products were obtained with sizes of 165, 300, 478, and 409 bp. TLR-4 gene of investigated Murrah buffaloes was highly polymorphic with AA, AB, and BB genotypes as revealed by PCR-RFLP analysis using *Dra I*, *Hae III*, and *Hinf I* REs. Nucleotide sequencing of the amplified fragment of TLR-4 gene of Murrah buffalo was done. Twelve SNPs were identified. Six SNPs were nonsynonymous resulting in change in amino acids. Murrah is an indigenous Buffalo breed and the presence of the nonsynonymous SNP is indicative of its unique genomic architecture. Sequence alignment and homology across species using BLAST analysis revealed 97%, 97%, 99%, 98%, and 80% sequence homology with *Bos taurus*, *Bos indicus*, *Ovis aries*, *Capra hircus*, and *Homo sapiens*, respectively.

## 1. Introduction

India is of a fortune position of having the world's best breeds of buffaloes for milk production. Special attention has to be focused on Murrah breed of Buffalo whose breed average milk production is about 2200 kg per lactation. Buffalo contribute more than fifty percent milk to the total milk produced in India. However, due to increased prevalence of infections, the realization of their true genetic merit has been hampered. Among infectious diseases, mastitis, an inflammatory disease of the mammary gland generally caused by intramammary infections, is the most common, costly, and devastating disease in dairy animals. Therefore, attention needs to be focused to study the genes involved in disease resistance, especially for mastitis. Genes associated with immune responses of the mammary gland are potential markers because of their importance in mastitis. The toll-like receptor-4 (TLR-4) is an important pattern recognition receptor that recognizes endotoxins associated with gram negative bacterial infections [1, 2]. Its role in pathogen recognition and subsequent initiation of the inflammatory and immune responses, and highly polymorphic nature in the bovine species, make it a suitable candidate gene for use in marker-assisted selection for enhancing disease resistance in dairy animals [3]. The TLR-4 gene coding region is 2526 bp long consisting of 3 exons and is located on chromosome BTA 8. Bovine TLR4 has three exons, exon 1 includes coding base pairs 1–95, exon 2 consists of base pairs 96–260, and exon 3 comprises base pairs 261–2526. The whole genomic length is estimated to be approximately 11 kb, of which the first intron comprises about 5 kb and the second is 3 kb. Polymorphic studies and nucleotide sequencing of TLR-4 gene have been reported in cattle [4, 5]. With the exception of the thesis by Sonawane [6], no such information is available in Murrah buffalo. Considering the importance of Murrah buffalo in milk production, the present study was undertaken to partially sequence the buffalo TLR-4 gene and to detect SNP.

## 2. Materials and Methods

### 2.1. Experimental Animals and Sampling

The animals included in the present study were from the herd of Murrah Buffaloes maintained at cattle yard of National Dairy Research Institute, Karnal, Haryana, India. Blood samples were collected from 102 randomly selected lactating animals.

### 2.2. Isolation of Genomic DNA

Ten mL of blood was collected aseptically by jugular vein puncture in a sterile vacutainer tube containing 15% of 0.12 mL EDTA solution (Becton-Dickinson vacutainer). The samples were transported to the laboratory in an icebox and stored at 4°C till further processing for DNA isolation. The blood samples were centrifuged and DNA was isolated from the buffy coat alone using phenol-chloroform method, as described by Sambrook et al. [7] with few modifications.

### 2.3. Quality, Purity, and Concentration of DNA

Quality of DNA was checked by electrophoresis by loading 2 *μ*L DNA on 0.8% agarose in horizontal minielectrophoresis unit using 1xTBE as running buffer at 30–40 volts for about one and a half hours. After electrophoresis, the gel was stained with ethidium bromide solution (0.5 *μ*g/mL). The gel was photographed by Gel Documentation System and files stored.

Quality and quantity of DNA was estimated by spectrophotometer method. DNA (2 *μ*l) was dissolved in 98 *μ*l of double-distilled water and loaded into a 100 *μ*l cuvette. Optical density (OD) was determined at wavelengths 260 nm and 280 nm in a UV-Vis spectrophotometer against distilled water as blank sample. The ratio between OD_260_ and OD_280_ was calculated. The sample possessing a ratio of less than 1.7 and more than 2.0 was subjected to proteinase K digestion and DNA extracted with phenol chloroform isoamyl alcohol as described previously.

### 2.4. PCR-RFLP of TLR4 Gene

The primer pairs for exons 1 and 2 of TLR-4 gene were designed by using the primer 3 plus software, and primers 3 and 4 which are part of exon 3 were used as described by Sonawane [6]. Primers for TLR 4 Gene are as follows: Forward 5′-CATGCTGATGATGATGGCGCGTG-3′ and Reverse 5′-CGTACGATCACTGTACGCAAGG-3′ for exon 1, Forward 5′-TTGTTCCTAACATTAGTTACC-3′ and Reverse 5′-CTGGATAAATCCAGCACTTGCAG-3′ for exon 2, Forward 5′-GGCTGGTTTTGGGAGAATTT-3′ and Reverse 5′-TGTGAGAACAGCAACCCTTG-3′ for exon 3.1, and Forward 5′-CCAGAGCCGATGGTGTATCT-3′ and Reverse 5′-CACTGAATCACCGGGCTTT-3′ for exon 3.2.

For amplification, 25 *μ*L of PCR reaction was prepared by adding each primer, dNTPs, MgCl_2_, 10× PCR assay buffer, DNA template, and *Taq DNA* polymerase. The amplification was carried out using a preprogrammed thermal cycler (Eppendrof Mastercycler) with the following conditions: initial denaturation of 2 min at 95°C followed by 35 cycles of denaturation at 94°C, annealing at 55°C for primers 1 and 2, 54°C for primers 3 and 4 for 30 sec, extension at 72°C each of 1 min 30 sec and lastly the final extension of 7 min at 72°C. After PCR amplification, 5 *μ*L the PCR product was checked on a 1.5% agarose gel to verify the amplification of target region.

The amplified PCR fragments, namely, exon 1, exon 2, exon 3.2, and exon 3.2 of TLR 4 gene were digested with *Dra I* (5′*⋯*TTT/AAA*⋯*3′), *Hae III* (5′*⋯*GG/CC*⋯*3′), *Hind III* (5′*⋯*A/AGCTT*⋯*3′), and *Hinf I* (5′*⋯*G/ANTC*⋯*3′) restriction enzymes, respectively. The reaction mixture (20 *μ*L) for each enzyme was kept for incubated at 37°C for 4 hours. Restriction fragments were resolved on 2-3% agarose gel horizontal electrophoresis and visualized by ethidium bromide staining. The ethidium bromide was added to the agarose gel of 1 *μ*L/100 mL of gel. The agarose gel electrophoresis was performed in 1X TBE buffer at 100 volts for 30, 60, and 90 minutes till complete separation and visualization of all fragments of RE-digested gene fragments, DNA ladder and PCR marker. The restriction-digested gene fragments were visualized on UV transilluminator and photographed with gel documentation system.

## 3. Custom DNA Sequencing

Amplified PCR products were subjected to custom DNA sequencing from both ends (5′ and 3′ ends). Representative samples from each of the variants obtained by RFLP analysis were also custom sequenced (Chromous Biotech Pvt. Ltd., Bangalore, India). Nucleotide sequences were visualized using Chromas (Ver. 1.45, http://www.technelysium.com.au/chromas.html). Sequence data were edited using the Editseq program, and multiple sequence alignments were performed with MegAlign program of LASERGENE software, respectively (DNASTAR, Inc, Madison WI, USA). The forward and reverse sequences for each PCR fragment were assembled to form contigs of the respective region. The TLR-4 gene sequence of Murrah was compared with that of *Bubalus bubalis* (EU386358) sequence to annotate different exonic regions putatively to identify SNPs in respective region. The partial coding DNA sequence of bubaline TLR-4 gene (exons 1, 2, and 3) was conceptually translated and compared with that of the *Bubalus bubalis* to detect amino acid changes in buffalo TLR-4 regions included in present study. The contiguous TLR-4 nucleotide sequence was subjected to Basic Local Alignment Search (BLAST) at NCBI database to determine the sequence homology with the corresponding regions of other species.

## 4. Results and Discussions

The sample genomic DNA was amplified by Polymerase Chain Reaction (PCR). PCR conditions were standardized. The amplified PCR product was checked on 1.5% agarose to verify the amplification of target region. The amplified sizes were estimated as 165 bp for exon 1, 300 bp for exon 2, 478 bp for exon 3.1, and 409 bp for exon 3.2.

Polymerase Chain Reaction-Restriction Length Polymorphism (PCR-RFLP) analysis of each PCR product was carried out using *Dra I, Hae III, Hind III*, and *Hinf I* restriction enzymes for all 102 animals included in this study system. Restriction digestion of amplicon of Exon 1 revealed two fragments of 110 and 55 bp exhibiting monomorphic (BB) pattern in all the animals under study. However, exon 2 of TLR4 gene did not have any cutting site with *Dra I*. Restriction digestion of exon 3.1 resulted in resolution of 5 fragments, identified as AA (478, 350, 272, 169 bp), AB (478, 350, 272, 169, 74 bp), and BB (272, 169, 74 bp) genotypes. TLR4-exon 3.2 exhibited AA (409 bp) AB (409, 246,163 bp) and BB (246,163 bp) genotypes with this restriction enzyme.

PCR-RFLP of exon 1 with *Hae III *RE yielded two genotypes AB (165, 122, and 43 bp) and BB (122 and 43 bp). Exons 2 and 3.1 of TLR4 gene did not have any cutting site with *Hae III RE. *Exon 3.2 exhibited AA (409 and 309 bp), AB (309, 200, 142, and 100 bp), and BB (200, 142, and 100 bp) genotypes.

PCR-RFLP analysis of TLR4 gene using* Hind III *restriction enzyme did not reveal any cutting site.

PCR-RFLP analysis of exon 1 of TLR4 gene using* Hinf I *restriction enzyme yielded two fragments of 110 bp and 55 bp size. No polymorphism was found with respect to *Hindf I *RE. Exon 2 of TLR4 gene did not have any cutting site with *Hinf I *RE. The only genotype exhibited by exon 3.1 of TLR-4 was BB with 291 and 187 bp restriction fragment size. For exon 3.2, genotypes with restriction fragment were identified as AA (409, 308, and 292 bp), AB (308, 292, 200, 136, and 100 bp), and BB (200, 136, and 100 bp).

The present findings of Murrah buffalo could not be compared with other studies, as no such report on buffalo is available in the literature. In a recent study by Sonawane [6] in the same buffalo herd, three genotypes AA, AB, and BB with variable frequencies using Alu I, Bsp 1286 I, and BsHKAI restriction enzymes were reported. However, exon 2 in that study was also observed as highly conserved part of the gene. Hence, no cutting site was observed using 7 enzymes (3 REs by Sonawane [6], and 4 in the present study). Sharma et al. [4] reported CC, CG and GG, genotypes in the promoter region (P 226) of Holstein cattle. Wang et al. [5] reported moderate occurrence of polymorphism with AluI in Chinese Simmental, Holstein, and Sanhe cattle. 

## 5. Analysis of Sequencing Data

**Figure 1:**
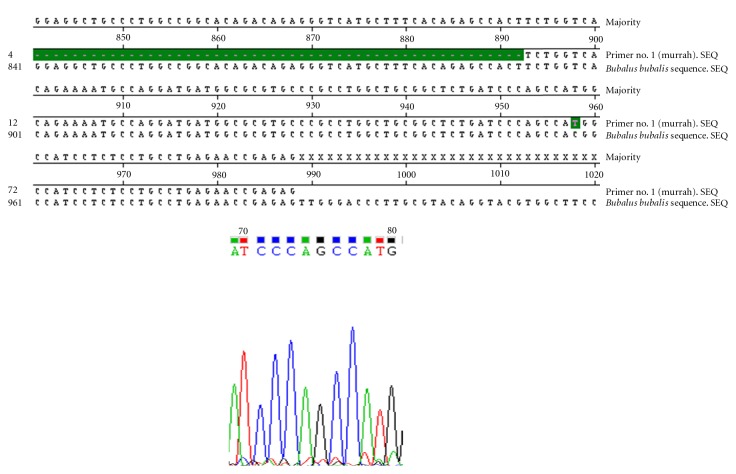
Clustal W alignment and chromatograph of exon 1 of TLR-4 gene in Murrah.

**Figure 2:**
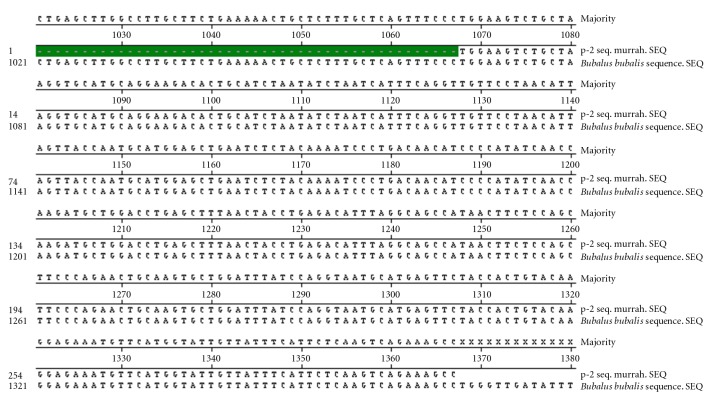
Clustal W alignment and chromatograph of exon 2 of TLR-4 gene in Murrah.

**Figure 3:**
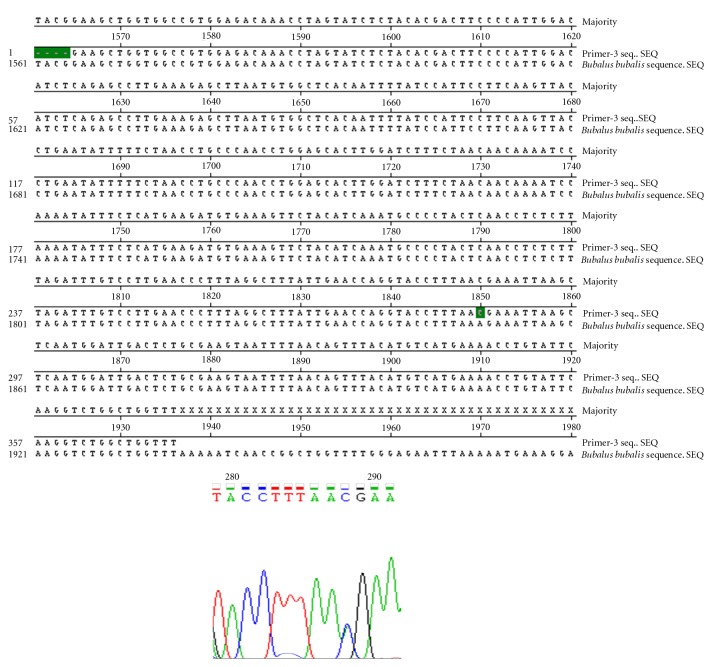
Clustal W alignment and chromatograph of contig 3.1 of TLR-4 gene in Murrah.

**Figure 4:**
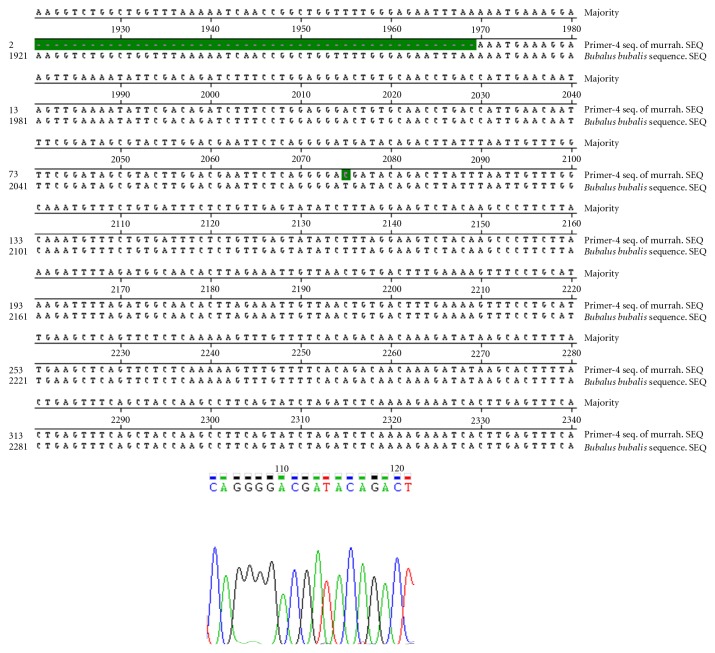
Clustal W Alignment and Chromatograph of Contig 3.2 of TLR-4 Gene in Murrah.

**Figure 5:**
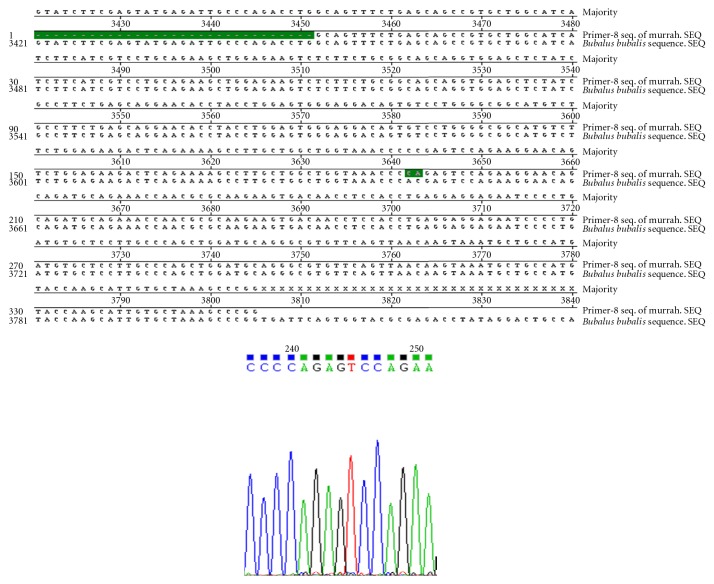
Clustal W alignment and chromatograph of contig 3.2 of TLR-4 gene in Murrah (Contd.).

**Figure 6:**
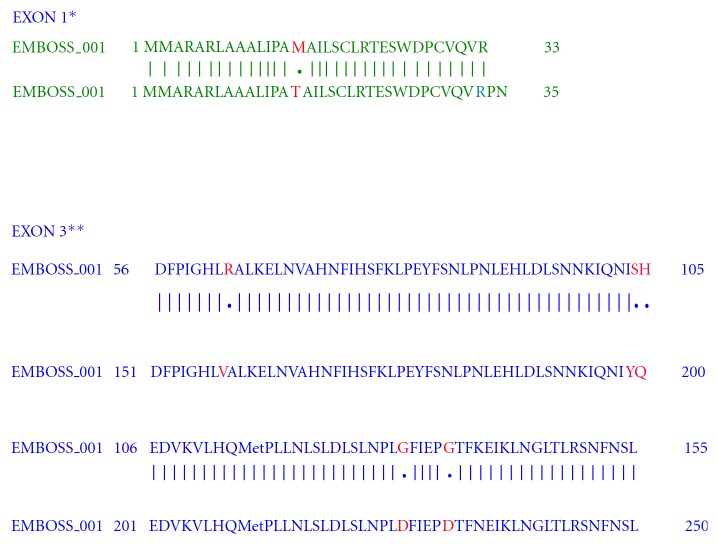
Multiple alignment of conceptualized TLR4 amino acid sequences of *Bubalus bubalis* (accession number EU 386358) and present study. ∗In exon 1 amino acid substitution: threonine (T) to methionine (M). ∗∗In exon 3 amino acid substitution: valine (V) to arginine (R), tyrosine (T) to serine (S), glutamine (Q) to histidine (H), and aspartic acid (D) to glycine (G).

Nucleotide sequencing of amplified fragments of TLR-4 gene of buffalo was performed (Figures 1, 2, 3, 4, and 5). The Coding DNA Sequence of bubaline TLR4 gene compared with that of this sequence was compared to the reported sequence of *Bubalus bubalis *with NCBI accession number EU386358. The sequence obtained for Murrah was compared and aligned custom sequenced using the MegAlian program of DNASTAR software. Amplified regions of the 4 contig regions were custom sequenced by using forward and reverse primers. Sequence data were analysed using chromas (Ver.1.45, http://www.technelysium.com.au/chromas.html). Clustal W multiple alignments with *Bubalus bubalis* sequence revealed a total of 12 bp changes, one in exon 1 and 11 in exon 3. Multiple alignment revealed a total of 12 mutations: 1 in exon1 and 11 in exon 3. Out of these 12 mutations, six were nonsynonymous resulting in change in Threonine to Methionine, Valine to Arginine, Tyrosine to Serine, Glutamine to Histidine, and Aspartic Acid to Glycine (at two positions) (Figure 6).

## 6. SNP Identification

Sequence analysis revealed 12 SNPs in the coding (exonic) region of TLR-4 gene given in Table 1. The Coding DNA Sequences of Murrah TLR-4 gene (Exon 1, 2, and 3) were conceptually translated and compared with those of *Bubalus bubalis *reported sequences (NCBI Accession number EU386358). At position 75nt of exon 1, only one SNP (T to C) has been identified which has resulted in a substitution of Threonine to Methionine. Exon 2 did not show any change in base sequence. However, a total of 11 SNPs have been identified in exon 3 at nucleotide positions 311, 315, 316, 318, 386, 401, 411, 551, 555, 636, and 994. Only five of these nucleotide changes result into changes in amino acids leading to nonsynonymous SNPs. In the only report available till date, Sonawane [6] has reported a total of six SNPs, out of which 4 are nonsynonymous, two each in exons 1 and 3. He also did not observe any SNP in exon 2. However, in Holstein cattle, Sharma et al. [4] have reported 3 SNPs: 1 in promoter region (P-226) and 2 in exon 3 (1656 and 2021). Wang et al. [5] identified 1 SNP at nucleotide 1397 in exon 3. Wang et al., 2007 have reported 31 SNPs scattered through the 5′ flanking region to exon 3. Five of these SNPs were coded for amino acid substitution.

## 7. Sequence Alignment and Homology Across Species

The contiguous TLR-4 nucleotide sequence was subjected to basic local alignment search tool (BLAST) at NCBI database to know the sequence homology with the corresponding regions of other species. It revealed 97%, 97%, 99%, 98%, and 80% homology with *Bos indicus, Bos taurus, Ovis aries, Capra hircus, and Homo sapiens *respectively. Sequence alignment and homology across species using Basic Local Alignment Search Tool (BLAST) analysis revealed 97%, 97%, 99%, 98%, and 80% sequence homology with *Bos taurus, Bos indicus, Ovis aries, Capra hircus, *and *Homo sapiens*, respectively. 

## 8. Conclusion

In conclusion, nucleotide sequencing of the amplified fragment of TLR-4 gene of Murrah buffalo revealed Twelve SNPs: 1 in exon1 and 11 in exon 3. Six SNPs were nonsynonymous resulting in change in amino acids. Murrah is an indigenous Buffalo breed, and the presence of the nonsynonymous SNP is indicative of its unique genomic architecture. Sequence alignment and homology across species using Basic Local Alignment Search Tool (BLAST) analysis revealed 97%, 97%, 99%, 98%, and 80% sequence homology with Bos taurus, Bos indicus, Ovis aries, Capra hircus, and Homo sapiens, respectively. 

## Figures and Tables

**Table 1:** SNPs identified in TLR-4 gene (Murrah buffaloes).

Region	Position	Base change	Amino acid substitution
Exon 1	75	C–T	T–M

Exon 3	311	A–C	R–V
	315	A–C	S–Y
	316	T–C	—
	318	A–C	H–Q
	386	C–A	G–D
	401	G–A	G–D
	411	A–C	—
	551	T–G	—
	555	C–A	—
	636	T–C	—
	994	A–G	—

T: Threonine; M: Methionine; R: Arginine; V: Valine; S: Serine; Y: Tyrosine

H: Histidine; Q: Glutamine; G: Glycine; D: Aspartic Acid;

—: means that there was no amino acid substitution.
